# A systems biology approach towards the prediction of ciliopathy mechanisms

**DOI:** 10.1186/2046-2530-4-S1-P88

**Published:** 2015-07-13

**Authors:** K Koutroumpas, J Van Dam, G Toedt, Q Lu, J Van Reeuwijk, K Boldt, E.U. The Syscilia Consortium, T Gibson, R Roepman, M Ueffing, RB Russell, M Huynen, M Elati, F Képès

**Affiliations:** 1 Institute of Systems and Synthetic Biology, Genopole, CNRS, University of Evry, Evry, France; 2 Centre for Molecular and Biomolecular Informatics, Radboud University Nijmegen, Nijmegen, The Netherlands; 3 European Molecular Biology Laboratory, University of Heidelberg, Heidelberg, Germany; 4 Cell Networks, Bioquant, Cluster of Excellence, University of Heidelberg, Heidelberg, Germany; 5 Radboud Institute for Molecular Life Sciences, Radboud University Nijmegen, Nijmegen, The Netherlands; 6 Department of Human Genetics, Radboud University Medical Center, Radboud, The Netherlands; 7 Division of Experimental Ophthalmology and Medical Proteome Center, Eberhard-Karls Universität Tübingen, Tübingen, Germany; 8 Syscilia, Radboud Institute for Molecular Life Sciences, Radboud University Nijmegen, Nijmegen, The Netherlands

## Objective

Understanding the molecular and cellular mechanisms of ciliopathies is vital for dissecting their pathogenesis, identifying appropriate therapeutic targets and designing effective treatments. Recent advances in DNA sequencing technology have provided a torrent of genetic data that can now be used to elucidate the genetic basis of human diseases. A consensus has emerged among biologists that to fully exploit the available data, they have to be correlated with additional research. This is especially important for rare-disease genetics, because of the small number of available patients.

## Methods

Computational methods could be employed to tackle this problem. Integration of diverse biological data can provide additional evidence to support experimental observations. We present a statistical method that predicts disease causing genes by integrating protein interaction data and clinical phenotype annotations. For a given protein in the network the method predicts the clinical phenotypes that may appear upon protein alteration based on the phenotypes associated with the neighbours of the protein.

## Results

Application to protein interaction data from the SYSCILIA project (http://syscilia.org) and phenotype annotations from the HPO project (http://www.human-phenotype-ontology.org) succesfully predicts candidate Nephronophthisis genes. The results also imply that abnormal Hh signaling may be the cause of Nephronophthisis and GPCR mislocalization a possible way by which cilia defects affect Hh signaling.

## Conclusion

The results indicate that the developed method can be useful for the dissection of disease pathogenesis by predicting disease genes and drawing new hypotheses on the underlying mechanisms. Such hypotheses can assist in the design of new targeted experiments.

